# Root Canal Treatment and Filling

**Published:** 1917-04

**Authors:** Samuel M. Hinman


### ROOT CANAL TREATMENT AND FILLING

BY SAMUEL M. HINMAN, D.D.S.

Root canal treatment and filling is the subject which is more in the minds of the profession today than any other, and probably more has been written about it in the last few years than in the whole history of the profession previously.

The writer of a paper, no matter what methods he may employ or advocate, is confronted by a host of critics, some able and some not so able. I refer especially to the article by Dr. Grove in the January *Cosmos,* and to Dr. Price's talk at the Second District Society of New York, in Brooklyn on January 8.

Dr. Grove's article should serve to warn us of the dangers of the use of sulphuric acid and sodium and potassium in opening our root canals, but should not discourage us in their intelligent use. His experiment with formalin was probably in healthy root canals, and if it was not, how did he know that the injuries noted were caused by the formalin and did not exist previously? This should teach us, however, that formalin should not be used except in putrescent canals, and should not be employed in root fillings.

His statement in regard to the rarefaction of the tissues around the apices of the teeth which had been filled through the end, is as suggested by Ottolengui, without value, because it gives no previous history as to whether they were putrescent or whether vital pulps were removed, nor does it give any idea of the conditions of asepsis under which the filling operations were performed.

Dr. Price's statements that root canals once infected can not be sterilized by any known method, in the mouth, and that our chloropercha or guttapercha root canal fillings do not seal the roots and prevent further egress of bacteria through the apices, must be weighed against the results which have followed the treatment of infected roots by ionization and apioectomy. There are many cases on record of cures of joint infections, heart lesions, neuritis and tic douloureux, which have apparently resulted from the treatment, without extraction, of these so-called granulomas.

Asepsis in the handling of pulpless teeth is generally acknowledged to be of the first importance, and to obtain this successfully, the rubber dam is most essential. The manufacturers make no pretense that this article is sterile, and it therefore becomes our duty to render it so before placing it in the mouths of our patients. The writer's method (1) is to cut the rubber in suitable sizes, wash and scrub with soap and water, and then boil for ten minutes. The pieces are then dried between sterile towels, without touching the hands, and are then placed between pieces of sterile gauze in a glass jar, to be used as needed. The teeth should then be wiped with a pledget of cotton saturated with hydrogen dioxid. The rubber is then placed over the tooth, the gums having been painted with iodin, taking in, if possible, at least one tooth on each side of the one to be treated, and is held in place by means of a clamp, ligature, or sometimes copper wire. The teeth and adjoining rubber are then wiped off with phenol followed by alcohol, and we are ready to operate. The teeth offering the greatest difficulty are the molars, with nearly parallel sides, where the clamp slips off. This may sometimes be obviated by holding the clamp in place, drying the tooth with alcohol and hot air, and painting with a varnish solution. This, when dry, will usually prevent clamp from slipping.

Any tooth in the mouth to which a band can be fitted can be placed under the rubber. The Hoods copper bands will be found very convenient for this purpose, as they come in all sizes and may be fitted and cemented very readily, the pulp chamber having been filled with temporary stopping to keep the cement from the cementation or the band from interfering with our root canal work. If necessary the gum is first packed away with gutta-percha.

In infected roots, or in roots which have been previously treated and filled with pastes or cotton and creosote, the first treatment consists of opening into the pulp chamber (without attempting to enter the root canals) and sealing in with temporary cement, a dressing of cresol seventy-five parts, formalin twenty-five parts, for twenty-four to forty-eight hours. The cases in the writer's experience most likely to develop acute pericementitis following this treatment, are the ones which have been filled for years with cotton fillings, and have never given any outward signs of trouble. Two treatments of formocresol before cleaning out the roots have failed to prevent the development of acute symptoms.

Only large round burs should be used within the pulp chamber, as inverted cones will cut a ledge in the cavity and afterwards interfere with our entrance into the canals. Any sacrifice necessary must be made in the crown of the tooth, in order to obtain a direct access into the canals. Failure to do this is a most frequent cause of inability to reach the apex. Root canal drills in the engine should not be used. They might be permissible once the apex has been reached, or to enlarge the orifice of a canal, but if forced forward, they will surely throw you off the proper route, and if you use one a hundred times and break it on the hundredth and first time, you will most certainly rue it.

Sodium and potassium is introduced into the canals only in case portions of the pulp remain which can not be removed by means of the broaches. It should be kept in mind that sodium and potassium acts by virtue of its great affinity for water, and therefore destroys any organic material whether living or dead, and should not be employed in large canals from which a vital pulp has been removed.

This reaction is called saponification, and its products, soaps, the potash forming the soft soap, and the sodium the hard soap. If this reaction is complete and within the canal, and no free alkalis are forced through, I can not see how there can be an injury to the apical tissues. The alkali may be neutralized by a fifty percent solution of sulphuric acid, and this is followed by a sodium bicarbonate solution.

The canals are then cleaned out and enlarged by means of the Kerr files. These little instruments are most effective for this purpose, and the method of using is to give them a half turn and then pull out, rotating between the finger and thumb. Sulphuric acid is employed for the purpose of enlarging very fine root canals, and assists principally by its action upon the salts of the dentine. This is introduced by means of a dropper, and worked into the canals with the acid proof broaches.

The files and broaches may be used several times, but too careful economy in their use should be guarded against, as one broken one in a root canal will cost you the equivalent of a whole gross of broaches.

Sulphuric acid, as pointed out by Grove, forms salts from the dentin which are insoluble in the acid itself, thus showing the futility of sealing the acid in the tooth, and also showing the necessity of working it in with broaches, and frequently neutralizing and washing out. Hydrochloric acid as recommended by Grove seems to work a little more rapidly than sulphuric acid, although it is therefore more dangerous, and a perforation can more readily result. It is also very difficult to keep in your cabinet without injury to the instruments.

All root canal instruments are kept on a tray in the formalin sterilizer. No dependence, however, is placed in this as a means of sterilization, but simply as a means of keeping them sterile once they have been sterilized by steam or by boiling.

The cotton used in wiping out canals is sterilized in small packages as suggested by Best. An ordinary vulcanizer is used as an autoclave. The gauze and cotton are placed in the vulcanizer with a very small amount of water in the bottom, and the temperature raised to three hundred degrees Fahrenheit and kept there for thirty minutes. It is then allowed to cool and the gauze removed with pliers and placed in the formalin cabinet.

When the end of the root has been reached or very nearly reached, ionization is employed, the method being as follows: The rubber dam in place, the canals are flooded with alcohol and dried with air in the root canal drier. A broach of chemically pure zinc is attached to the broad holder of the machine, and placed as far into the canal as possible, then the root is flooded, using an abscess syringe with a three percent solution of zinc chlorid, and a pledget of cotton saturated with the zinc chlorid is placed in the pulp chamber. The broach holder is connected to the positive pole, and the negative electrode (a moist sponge) is held in the hand of the patient on the same side as the tooth under treatment. The current is then advanced to one-half M. A., or if the patient can bear the current, it may be sometimes advanced to four M. A. The writer prefers using the hand for the negative electrode, as more current can be employed without discomfort, and it is more easily adjusted. This treatment is given each root canal from one to five times, depending on the condition under treatment. The treatments are given one week apart, although it may be used as often as twice a day in cases of acute pericementitis. The treatment of two or more canals at the same time has been advocated by some operators, but as the current goes along the line of least resistance, it could not be expected that the reaction would be equally effective in each canal.

The writer has had the following experience, not once but a dozen times. A tooth so sore that great difficulty is experienced in masticating, and considerable pain caused by adjusting rubber and removing treatment or filling—root canal ionized and soreness entirely disappears, sometimes at once, sometimes in a few hours, and sometimes only after another electrical treatment.

Sturridge of London has demonstrated that the zinc chlorid does penetrate the dentin by the following experiment: A solution of zinc carbonate is placed in a root canal of an extracted tooth, the positive pole in the canal and the negative pole in the electrolyte of sodium chloride is removed, the ferrisulphate washed out, and the tooth soaked in potassium ferrocyanid. Potassium ferrocyanid unites with zinc carbonate to form prussian blue. Longitudinal and cross sections of the tooth were made and examined. It was found that the fibres of Tomes lying within the dentinal tubulae were stained all the way to the cementum and some of the ferri salts had penetrated even into the cementum. Doctors Asch and Stern performed this experiment on a tooth in the mouth which was to be extracted.

The writer attributes the remarkable results which follow the employment of this method largely to the stimulation of the tissues which the positive pole of a galvanic current encourages. He also believes that this is the only method besides amputation which even offers any hope (Dr. Price to the contrary, notwithstanding), of restoring these septic roots to a healthy condition. The impossible, however, should not be attempted, and the intelligent employment of this method calls for radiographs of all cases.

Many drugs will destroy bacteria, but they all must come in direct physical contact with the microorganism in order to do so. So-called ionization encourages us in the belief that we can at least inhibit the bacterial proliferation, and at the same time encourage the activity of the phagocytes in fighting and carrying away the microorganisms, and in stimulating the secreting of ferments, which dissolve the detritus and also stimulate the activity of bone-forming cells.

The root having been cleaned and ionized, we are ready for our filling. The root filling instruments are kept on a separate tray in the formalin cabinet, and they consist of guttapercha points kept in alcohol; the large points are sterilized by boiling and the small one by formalin, two bottles of chlorapercha (a thick and a thin solution) the thin for finer canals and to dilute the thick solution, twelve canal pluggers (Detroit Ins. Co.), a pair of scissors, a pad of sterile gauze and the aseptic paper points put up by J. & J., and some very fine smooth broaches with handles. The canal is dried with the paper points and with the Evans root drier. The paper points are taken from the box and placed on the sterile gauze, also the guttapercha points. The chlorapercha is introduced into the canal with the fine broaches and pumped to the end. A radiograph is at hand which shows the length of the root. A small point is clipped off with the scissors and introduced into the canal with the pliers. It is then pressed into place with the pluggers, using the finest one first and larger ones as the filling progresses.

In the filling operation the very greatest care must be taken that the fingers do not come into contact with anything that goes into the root canal, and that no broach or instrument is laid on any bracket or anything which is not known to be sterile. The outside of the tooth, the adjoining teeth and the rubber should be regarded with suspicion.

In the removal of vital pulps, conductive anesthesia is the method certainly the best suited to asepsis extirpation. The objections to this method are the time required to obtain perfect anesthesia, and the uncertainty of results unless infiltration is resorted to, and the unpleasant symptoms which sometimes follow, due either to faulty technic or to the action of the suprenin. The writer's own results with this method have improved since taking more time in making the injection—a two percent solution in freshly prepared Ringer's solution is used.

Arsenic should seldom, if ever, be employed, and if it is used it should not be allowed to remain in the tooth for more than twenty-four hours. It certainly should never be used in children's teeth. There is no reason to believe that its destructive action will stop exactly where you want it to before reaching the apex of the tooth. So many other methods are at hand today that we should discard this drug entirely.

The quickest method in the writer's hands, and his favorite one, in case of a sound tooth from which the pulp is to be removed for bridgework (notwithstanding Dr. Chayes to the contrary), is as follows: Rubber in place, grind through enamel with a stone under water; tooth dried and wiped with phenol and alcohol; nasal inhaler in place, $N_2O$ & O anesthesia, with a medium round bur, drill to pulp; a one-twelfth gr. pellet neurocain; a drop of alcohol on a wisp of cotton; a little pressure with soft rubber; and by the time the assistant has removed the face piece, you have complete anesthesia of the pulp.

In case of a tooth where a cavity already exists, Buckley's desensatizing paste may be sealed in for twenty-four hours, and this will usually produce sufficient anesthesia for devitalization, and will also help sterilize any carious dentin. An exposure can then be obtained with very little pain, and the pulp removed by pressure anesthesia in the usual manner.

In case of an exposure with considerable decay present, the decay must first be removed or a dressing sealed in for a few days, in order to render the cavity as aseptic as possible, as otherwise bacteria will surely be forced down into the root canal and even beyond, and pericementitis result.

A radiograph should always be at hand before starting into the root canals. Another radiograph is taken when the canal is filled, sometimes several are required during the progress of the cleansing, and if not satisfactorily filled, the entire filing is removed, using chloroform and Kerr files, and the obstruction cleaned out and the root re-filled, when another picture is taken. Each canal should be taken up separately and treated and filled. This is especially necessary in the case cf the upper molars, as the palatine and buccal roots lie in divergent places and can not be correctly shown on the same film.

The question of protruding the filling through the apex is one upon which there is probably more disagreement than any other. It would seem, however, that there should not be any question of leaving any portion of infected material within the apex of the canal, and it is the writer's opinion that encapsulation of the apex should always be attempted in these cases. If not, then amputation or extraction is indicated. Of course, in case of the removal of a live and uninfected pulp, if thorough asepsis is followed, there is much evidence to support the theory that a small amount of the pulp at the apex will continue to hold its vitality uninfected for many years.

In taking up this kind of root canal work, one of the greatest problems is said to be the securing of proper fees to warrant expending the time required. Along this line I would suggest that the proper appreciation of its value must exist first in the mind of the dentist before he can successfully convince his patient.

The time required, on an average, to treat and fill each root canal, will be found to be nearly, if not quite three hours, and will require at least two radiographs for each. The writer disavows any intention of giving the impression that he has reached a satisfactory solution of this great problem, or even that he fills every root that passes through his hands.

From his own limited experience, the conclusions reached by the writer after a study of the writings of Ottolengui, Rhein, Price, Grove, Peeso, Buckley, Greaves,

Kells, et al., is that every dental operator who wishes to do the best root canal work possible should provide himself with and operate his own radiograph outfit. These operators who do not follow up all their work with radiographs should select as a filling material some form of antiseptic filling, such as iodoform and eucalyptol, to be used instead of chloropercha, with their points. The operation should then be considered as a temporary one, and should be explained to the patient as such. Formalin should not be incorporated in their filling material.—*New Jersey Dental Journal.*

